# Breast tumor specific mutation in GATA3 affects physiological mechanisms regulating transcription factor turnover

**DOI:** 10.1186/1471-2407-14-278

**Published:** 2014-04-22

**Authors:** Aleksandra B Adomas, Sara A Grimm, Christine Malone, Motoki Takaku, Jennifer K Sims, Paul A Wade

**Affiliations:** 1 Laboratory of Molecular Carcinogenesis, National Institute of Environmental Health Sciences, 111 T.W. Alexander Dr, 27709 Research Triangle Park, NC, USA; 2 Division of Intramural Research, National Institute of Environmental Health Sciences (NIEHS), Research Triangle Park, NC, USA

**Keywords:** Breast cancer, ChIP-seq, GATA3, Mutation, Transcription factor

## Abstract

**Background:**

The transcription factor GATA3 is a favorable prognostic indicator in estrogen receptor-α (ERα)-positive breast tumors in which it participates with ERα and FOXA1 in a complex transcriptional regulatory program driving tumor growth. *GATA3* mutations are frequent in breast cancer and have been classified as driver mutations. To elucidate the contribution(s) of *GATA3* alterations to cancer, we studied two breast cancer cell lines, MCF7, which carries a heterozygous frameshift mutation in the second zinc finger of *GATA3*, and T47D, wild-type at this locus.

**Methods:**

Immunofluorescence staining and subcellular fractionation were employed to verify cellular localization of GATA3 in T47D and MCF7 cells. To test protein stability, cells were treated with translation inhibitor, cycloheximide or proteasome inhibitor, MG132, and GATA3 abundance was measured over time using immunoblot. GATA3 turn-over in response to hormone was determined by treating the cells with estradiol or ERα agonist, ICI 182,780. DNA binding ability of recombinant GATA3 was evaluated using electrophoretic mobility shift assay and heparin chromatography. Genomic location of GATA3 in MCF7 and T47D cells was assessed by chromatin immunoprecipitation coupled with next-generation sequencing (ChIP-seq).

**Results:**

GATA3 localized in the nucleus in T47D and MCF7 cells, regardless of the mutation status. The truncated protein in MCF7 had impaired interaction with chromatin and was easily released from the nucleus. Recombinant mutant GATA3 was able to bind DNA to a lesser degree than the wild-type protein. Heterozygosity for the truncating mutation conferred protection from regulated turnover of GATA3, ERα and FOXA1 following estrogen stimulation in MCF7 cells. Thus, mutant GATA3 uncoupled protein-level regulation of master regulatory transcription factors from hormone action. Consistent with increased protein stability, ChIP-seq profiling identified greater genome-wide accumulation of GATA3 in MCF7 cells bearing the mutation, albeit with a similar distribution across the genome, comparing to T47D cells.

**Conclusions:**

We propose that this specific, cancer-derived mutation in *GATA3* deregulates physiologic protein turnover, stabilizes GATA3 binding across the genome and modulates the response of breast cancer cells to estrogen signaling.

## Background

Accumulation of somatic mutations is responsible for development of breast cancer, as 85% of affected women have no family history of the disease (http://www.breastcancer.org). Nearly 31,000 point mutations and small insertions or deletions (indels) in at least 170 previously reported and novel cancer genes have been implicated in the development of breast tumors [1]. Whole exome sequencing places the zinc-finger transcription factor *GATA3*, with a 10% frequency of alterations, among the top three (together with p53 (*TP53*) and phosphoinositide-3-kinase (*PIK3CA*)) mutation driver genes in breast cancer [1,2].

On the basis of mutation pattern, Vogelstein and colleagues [3] classify GATA3 as a tumor suppressor. Indeed, in mice xenograft studies GATA3 was positively correlated with survival and lack of metastasis [4]. However, it has been also postulated that GATA3 defines a distinct class of cancer genes that are differentiation factors rather than conventional tumor suppressor genes, which affect the malignant phenotype by enforcing differentiation [5-7]. Specifically, conditional deletion of GATA3 is not sufficient to promote malignant progression, and is not tolerated in early tumors [5,8]. GATA3 has been shown in mouse model of breast cancer to maintain tumor differentiation, suppress dissemination and inhibit metastasis [8,9]. While GATA3 has been intensively studied in the immune system, where it functions in development and differentiation of T-cells [10], it is also an essential regulator of mammary-gland morphogenesis and luminal-cell differentiation [11,12]. It is frequently up-regulated in breast cancer and has been identified as a favorable prognosis marker [13]. GATA3 is involved in a positive cross-regulatory loop with estrogen receptor-α (ERα) [14] where they both serve as markers for luminal breast cancer [15,16].

The interplay of GATA3, ERα, and FOXA1 has been a topic of multiple functional genomic studies. Kong and co-authors defined an enhanceosome consisting of co-localizing ERα-FOXA1-GATA3 which recruits RNA Pol II and p300 [17]. The triple conjoint binding sites are highly represented at the locations involved in frequent long-range chromatin interactions and associated with genes that are most responsive to estrogen. In turn, Theodorou and colleagues silenced GATA3 and observed a global redistribution of FOXA1 and p300 cofactors, and active histone marks prior to estrogen stimulation [18]. These global genomic changes alter the ERα-binding profile that subsequently occurs following estrogen treatment, demonstrating that GATA3 can act upstream of FOXA1 in mediating ERα binding by modulating enhancer composition.

Haploinsufficiency of GATA3 in humans results in HDR syndrome, a rare condition inherited as autosomal dominant trait, characterized by hypoparathyroidism, deafness, and renal dysplasia [19]. Genomic alteration of *GATA3* associated with HDR syndrome include large deletions removing the entire gene and flanking sequences, splice site mutations, indels, and point mutations resulting most often in frameshifts [20]. Mutations in HDR patients localized in the second zinc finger (ZnF2) of *GATA3* or adjacent amino acids result in loss of DNA binding, whereas those in the first zinc finger (ZnF1) lead to loss of interaction with a cofactor, FOG2, or altered DNA-binding affinity [20,21]. Interestingly, while HDR *GATA3* mutations are spread throughout the gene, breast cancer mutations cluster around ZnF2 and C-terminal domain [1,22,23]. Analysis of six different heterozygous *GATA3* mutations from eight breast tumors has demonstrated loss or reduction of DNA binding ability, aberrant nuclear localization, decrease in transcription activation, and alterations in invasiveness, but not proliferation [22]. However, it is unclear how those functional modifications contribute to the oncogenesis process in breast cancer.

The aim of the present study was to evaluate the effect of a breast cancer-specific mutation in *GATA3* on biochemical properties and genomic location of the protein. We utilized two luminal breast cancer cell lines, MCF7 harboring a heterozygous frameshift mutation in ZnF2, and T47D carrying wild-type version GATA3. We observed that mutant GATA3 was expressed at elevated levels relative to wild-type protein and it accumulated in nuclei. Surprisingly, the mutation led to enhanced protein stability following challenge with estrogen receptor agonist or antagonist. This increased stability led to increased levels, but not to global redistribution, of GATA3 binding in the genome as determined by ChIP-seq. The data collectively support the hypothesis that the carboxyl terminus of GATA3 contains protein regulatory information that ensures appropriate turnover following ligand binding by ERα.

## Methods

### Cell culture

Human breast carcinoma cell lines MCF-7 and T47D were obtained from the American Type Culture Collection (Manassas, VA, USA) and cultured in DMEM/F-12 medium supplemented with 10% FBS at 37°C in 5% CO_2_. Protein stability was evaluated in the normal growth medium and cells were treated with 1 μM cycloheximide (CHX) and/or 1 μM MG132 (MG) for up to eight hours. For estrogen starvation assays, cells were grown for 72 hours in MEM medium containing 5% FBS and then for 24 hours in phenol red-free MEM supplemented with 5% charcoal-dextran stripped FBS. Cells were treated with 50 nM 17β-estradiol (E2) for 24 hours. The effect of ERα inhibitor, ICI 182,780 (ICI) was tested in normal growth medium. ICI was added at 100 nM concentration and cells were harvested 24 hours later. MG (EMD Biosciences, San Diego, CA, USA) was dissolved in DMSO, CHX (Cayman Chemical, Ann Arbor, MI, USA) in water, ICI (Tocris Bioscience Ellisville, MS, USA) and E2 (Sigma, St. Louis, MO, USA) in ethanol.

### Subcellular fractionation

Cells were grown in 10 cm tissue culture dishes until they were 70-80% confluent. The cells were washed with PBS, collected by scraping and resuspended in buffer containing 0.15 M NaCl, 10 mM HEPES, pH 7.4, 1.5 mM MgCl_2_, 10 mM KCl, 0.5% NP-40, 0.5 mM DTT and protease inhibitors. The cytoplasmic fraction was separated by centrifugation at 2500 rpm for 10 min. The pellet was resuspended in nuclear extraction buffer containing 0.1, 0.2, 0.4 or 0.8 M NaCl, 25 mM HEPES, pH 7.4, 0.15 mM spermidine, 0.5 mM spermine, 5% glycerol, 1 mM EDTA and protease inhibitors. Samples were rotated for 30 min at +4°C and spun down in Optima Max centrifuge (Beckman Coulter, Brea, CA, USA) at 38,000 rpm for 45 min at +4°C. The nuclear fraction was collected and remaining pellet was dissolved in lysis buffer (8 M urea, 1% SDS, 0.125 M Tris, pH 6.8).

### Immunoblotting

Whole cell lysates were obtained using 8 M urea lysis buffer (8 M urea, 1% SDS, 0.125 M Tris, pH 6.8). Protein extracts (15 μg) were resolved on SDS–PAGE gels and immunoblotted using the following antibodies: GATA3 (D13C9; Cell Signaling Technology, Danvers, MA), FOXA1 (ab23738; Abcam, Cambridge, MA, USA), ERα (sc-543; Santa Cruz Biotechnology, Santa Cruz, CA, USA) and actin (ab8226; Abcam). Signal intensity was analyzed using rectangular volume tool in Quantity One Analysis Software (Bio-Rad, Hercules, CA, USA) with global background subtraction.

### Immunofluorescence staining

Cells were grown on glass coverslips in six-well tissue culture dishes. They were fixed with 4% formaldehyde in PBS for 10 min, washed with PBS, and permeabilized with 0.1% Triton X-100 for 2 min, washed with PBS, and blocked with 5% BSA in PBS. The coverslips were incubated with the anti-GATA3 antibody (Cell Signaling Technology) for one hour, washed with PBS, incubated with the secondary antibody (Alexa Fluor 568, Life Technologies, Grand Island, NY, USA) for one hour, washed with PBS, and mounted on glass slides with mounting medium containing 4′,6-diamidino-2-phenylindole (DAPI). The slides were examined and photographed using a Zeiss Axiovert 200 M microscope equipped with an Axiocam MR digital camera controlled by AxioVision software (Zeiss, Thornwood, NY, USA).

### Expression and purification of the DNA binding domain of GATA3

DNA binding domain (DBD) of GATA3 (amino acids 261 to 371) was cloned into the pET-15b vector to produce a hexahistidine tagged fusion protein. The expression vector was transformed into the *E.coli* BL21 (DE3) CodonPlus RIL cells, and the cells were cultured at 37°C. The bacterial cell lysate was centrifuged at 15,000 rpm for 20 min. The supernatant was mixed gently by the batch method with Ni-NTA beads (Qiagen, Valencia, CA, USA) at +4°C for 30 min. The beads were washed with 5 mM imidazole-containing buffer and GATA3-DBD was eluted with 500 mM imidazole-containing buffer. The fractions containing GATA3-DBD were subjected to MonoS column (GE Healthcare Life Sciences, Pittsburgh, PA, USA) chromatography. The binding domain was eluted with a 4-column volume linear gradient of 100–600 mM NaCl. The protein was further purified by Superdex 75 column (GE Healthcare) in a buffer containing 20 mM Tris–HCl pH 7.5, 0.3 M NaCl, 10% glycerol, 2 mM 2-mercaptoethanol, and 1 μM zinc sulfate. For the purification of GATA3 mutant (D336fs) DBD, the Ni-NTA beads were washed with the 20 mM imidazole-containing buffer. The fractions eluted from Ni-NTA beads were dialyzed against 20 mM Tris–HCl pH 7.5, 0.3 M NaCl, 10% glycerol, 2 mM 2-mercaptoethanol, and 1 μM zinc sulfate buffer, and concentrated with Amicon ultra-centrifuge filter (Millipore, Billerica, MA, USA).

### Electrophoretic mobility shift assay (EMSA)

GATA protein (0.5, 1, 2 or 4 μM for the wild-type protein and 0.25, 0.5, 1 or 2 μM for the mutant protein) was incubated with 30 μM of 20 bp dsDNA (GATA3 recognition motif-containing oligonucleotide AATGTCCATCT*GATA*AGACG or GATA3 recognition motif-lacking oligonucleotide AATGTCAAACT*TTTA*AGACG) in 10 μl of a reaction buffer (28 mM Tris–HCl pH 7.5, 1 mM dithiothreitol, 0.8 mM 2-mercaptoethanol, 120 mM NaCl, 4% glycerol, and 0.4 μM zinc sulfate). After 10 min incubation at 37°C, the samples were analyzed by polyacrylamide gel electrophoresis, and the bands were visualized by ethidium bromide staining. In the competitive DNA binding assay, wild-type and mutated GATA3 DBDs were used individually or mixed in equimolar proportion. The reactions were performed with 15 μM of 20 bp GATA3 motif-containing oligonucleotide and 23 bp GATA3 motif-lacking DNA (CACTTTTTAACGTAATTTACTCT).

### Heparin chromatography

T47D and MCF7 nuclear extracts were prepared as described above, using nuclear extraction buffer containing 0.4 M NaCl. The extracts were applied to a 1 ml HiTrap Heparin Sepharose (GE Healthcare Life Sciences). The column was eluted with a 10 ml linear gradient of NaCl concentration from 0.1 to 1 M in 20 mM Hepes, pH 7.9 containing 20% glycerol, 0.2 mM EDTA, 0.1 mM PMSF, and 0.5 mM DTT. Separated fractions were analyzed by Western blot directed against anti-GATA3.

### Chromatin immunoprecipitation (ChIP) analysis

GATA3 antibody was generated in rabbits using recombinant 6x histidine tag-fused GATA3 full-length wild-type protein. ChIP was performed as previously described [24] with the following modifications. T47D or MCF7 cells were cross-linked with 1% formaldehyde in DMEM F12 for 10 min at room temperature, quenched with glycine, and then sonicated using Bioruptor (Diagenode, Liège, Belgium) to generate 200 to 400 bp DNA fragments. Immunoprecipitation was performed with GATA3 serum, and normal rabbit serum (Santa Cruz Biotechnology, Dallas, TX, USA) was used as a control. The efficiency of the reaction was verified using SYBR-green (Bio-Rad) based Real-Time PCR and primers developed by Eeckhoute et al. [14] for GATA3 binding sites at ESR1 locus. Quantitation of precipitated DNA was done using a standard curve with 10, 1, 0.1, and 0.01% of input DNA.

### ChIP-seq library construction

DNA immunoprecipitated by GATA3 antibody in four to five individual reactions performed at the same time was pooled for T47D and MCF7 cells separately and purified using MinElute PCR Purification kit (Qiagen). Total 100 μg of ChIP or input DNA, quantified with Qubit Fluorometer (Life Technologies, Grand Island, NY, USA) and dsDNA High Sensitivity Assay kit (Life Technologies), was used for library construction with the help of TruSeq RNA Sample Preparation kit (Illumina, San Diego, CA, USA). The library was prepared following the manufacturer’s instructions, starting with the end repair step, and amplified with twelve PCR cycles. Two sets of libraries (ChIP and input) were prepared for each of the cell lines from samples immunoprecipitated on separate occasions. The libraries were sequenced on a Genome Analyzer IIx (Illumina) as single end 36mers.

### ChIP-seq data analysis

To ensure that low quality reads were excluded from the analysis, the raw sequence reads were filtered to remove any entries with a mean base quality score < 20. Filtered reads were aligned to the human genome (Genome Reference Consortium build 37/hg19; excluding haplotype chromosomes) via Bowtie (v0.12.8 with parameters –m 1 –v 2) [25]; only reads that were mapped to an unambiguous ‘best’ genomic location with no more than two mismatches were accepted. To limit PCR amplification bias, duplicate reads were removed using MarkDuplicates.jar from the Picard tools package (v1.62) (http://picard.sourceforge.net). Replicate libraries were in good agreement and were merged prior to downstream analysis. All alignments were extended at the 3’ end to a length of 180 bases (the average expected genomic fragment size for these libraries). ‘bedGraph’ files were generated from these uniquely-mapped, non-duplicated, extended reads for visualization of aggregate genomic coverage. Peak calling for regions of enriched GATA3 binding was performed with HOMER (v4.1; with default parameters and “-style factor -tbp 0 -inputtpb 0”) [26] using input (unchipped) data to model background.

### Data release

GATA3 ChIP-seq data have been deposited in NCBI’s Gene Expression Omnibus [27] and are accessible through GEO Series accession number GSE51274 (http://www.ncbi.nlm.nih.gov/geo/query/acc.cgi?acc=GSE51274).

## Results

### Heterozygous mutation is present in *GATA3* gene in MCF7 cell line

**Figure 1:**
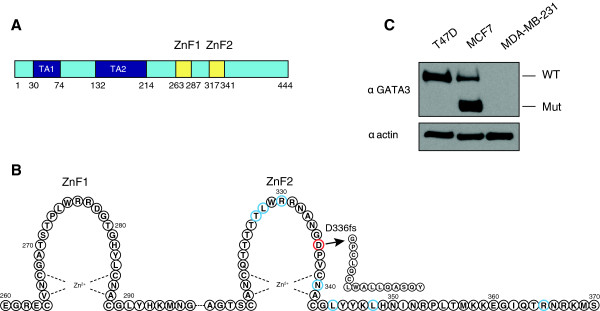
**Structure of GATA3 transcription factor. A)** GATA3 consists of 444 amino acids, which contain two transactivation domains (TA1 and TA2) and two zinc fingers (ZnF1 and ZnF2). **B)** The second zinc-finger binds to the canonical GATA motif, WGATAR, and the residues marked in blue are responsible for contact with DNA [10,32]. MCF7 breast cancer cell line carries a heterozygous mutation, which leads to a frameshift (D336fs) in the second zinc finger and synthesis of a truncated protein. Figure modified from Ali et al. [21] and Ho et al. [10]. **C)** Expression of wild type (WT) and mutated (Mut) GATA3 in MCF7 and T47D cells. MDA-MB-231, breast cancer cell line, negative for GATA3 was used as a control.

The human *GATA3* gene consists of six exons, encoding a protein of 444 amino acids, which contains two transactivation domains (TA1 and TA2) and two zinc fingers (ZnF1 and ZnF2) (Figure 1A). This gene is frequently mutated in breast tumors. The luminal breast cancer cell line MCF7 carries a heterozygous insertion at position 1566, which leads to a frameshift (D336fs) in the second zinc finger and synthesis of a truncated GATA3 protein [23] (Figure 1B). We confirmed the presence of guanine insertion in *GATA3* gene in our MCF7 stock and used a second luminal breast cancer cell line, T47D, wild-type for *GATA3*, as a control for our experiments. While T47D cells expressed only wild-type GATA3 protein (approximately 48 kDa), MCF7 cells contained both the full length GATA3 as well as a truncated protein of approximately 37 kDa (Figure 1C). Steady state levels of this truncated protein in MCF7 cells were significantly higher than the full-length GATA3 in the same cells (Figure 1C).

### Truncated GATA3 protein is easily released from the nucleus

**Figure 2:**
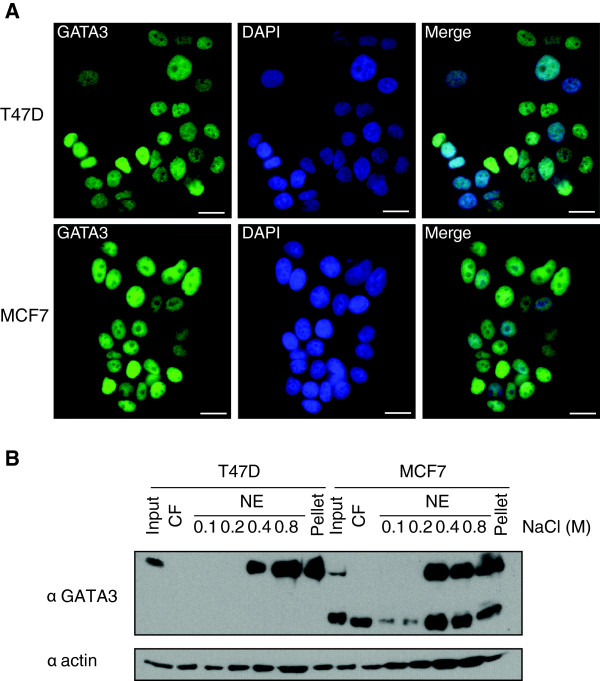
**Cellular localization of wild-type and mutated GATA3 in breast cancer cells. A)** Immunofluorescence staining of T47D and MCF7 cells. Scale bar corresponds to 20 μm. **B)** Subcellular fractionation of T47D or MCF7 cell extracts in 0.1-0.8 M NaCl. Input – whole cell lysate, CF – cytoplasmic fraction, NE – nuclear extract.

We used T47D and MCF7 cell lines to study the effect of the frameshift mutation in *GATA3* gene on the properties of the protein. Immunofluorescence staining was employed to verify cellular localization of GATA3 in T47D and MCF7 cells, demonstrating nuclear localization, regardless of the mutation status (Figure 2A). Subcellular fractionation with extraction buffer containing between 0.1 and 0.8 M NaCl, demonstrated that wild-type GATA3 was extracted efficiently from nuclei at moderate salt concentration (0.4 M NaCl) from nuclei in T47D cells (Figure 2B). The full-length protein behaved in a similar manner in MCF7 cells and was released from nuclei at 0.4 M NaCl. However, the truncated GATA3 was released from the chromatin with extraction buffer containing even the lowest NaCl concentration (0.1 M). The truncation mutant was present in the cytoplasmic fraction as well, suggesting that a pool of mutant protein is nuclear, but has impaired interaction with chromatin and is easily released from the nucleus.

### The second-zinc finger frameshift mutation stabilizes GATA3 protein

**Figure 3:**
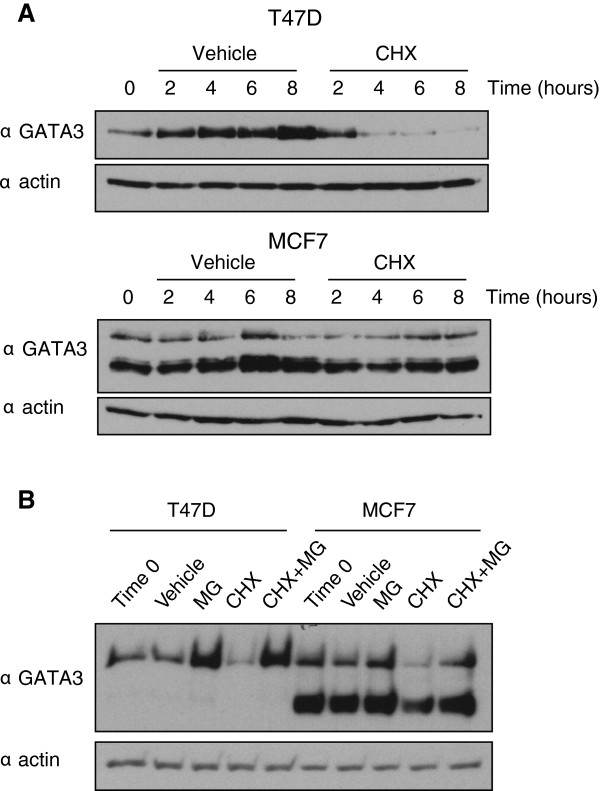
**Protein stability of wild-type and mutated GATA3. A)** T47D and MCF7 cells were treated with cycloheximide (CHX) or DMSO (Control) and collected at 2, 4, 6 and 8 hours. **B)** T47D and MCF7 cells were treated with cycloheximide (CHX), MG-132 (MG), or DMSO (Control) and collected 8 hours later.

The increased steady-state abundance of the truncated GATA3 mutant in MCF7 (Figure 1C, also [23]) suggested that the mutation impacts stability. To test this hypothesis, T47D and MCF7 cells were treated with a translation inhibitor, cycloheximide and GATA3 abundance was measured over time using immunoblot of whole cell lysates. Over the course of eight hours, levels of wild-type GATA3 in T47D cells decreased, with a significant reduction visible four hours after the treatment (Figure 3A, Additional file 1: Figure S1). In contrast, both wild-type and mutant GATA3 in MCF7 exhibited greater stability, with half lives in excess of 8 hours (Figure 3A, Additional file 1: Figure S1), suggesting that the mutant protein titrates out a factor integral to GATA3 turnover.

Protein stability controlled by action of the 26S proteasome is integral to the biology of ERα [28], which is found in close proximity to GATA3 at many genomic locations in breast cancer cells [17,18]. Inhibition of ubiquitin-proteasome pathway stabilizes GATA3 in developing T cells [29]. To determine whether GATA3 protein turnover is regulated in a similar, proteasome-dependent manner in breast cancer, we treated cells with cycloheximide and a proteasome inhibitor, MG132. In both T47D and MCF7 cells, proteasome inhibition alleviated the effect of translation inhibition on wild-type and, to a lesser degree, mutated GATA3 (Figure 3B). These data indicate that GATA3 is regulated at the protein level by the proteosome and that the cancer-specific mutation results in increased protein stability.

### GATA3 mutation uncouples turnover from the hormone response

**Figure 4:**
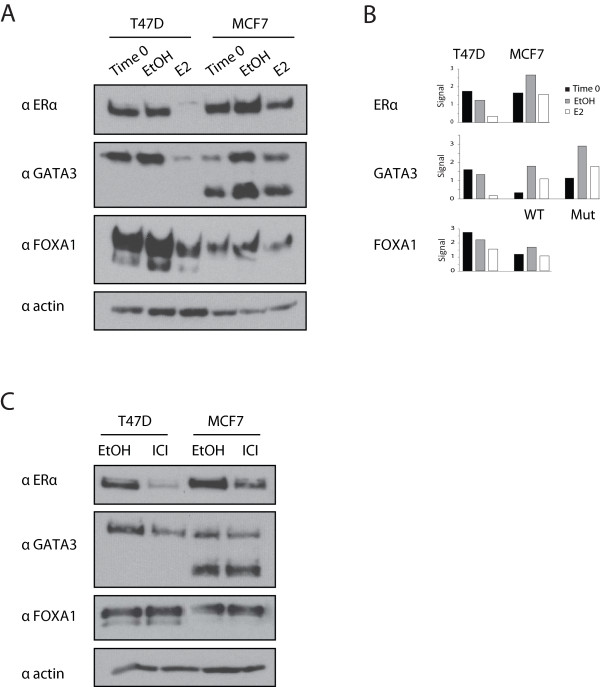
**Protein stability of GATA3 in cells treated with ERα agonist or antagonist. A)** T47D and MCF7 cells were grown in hormone-depleted conditions and then treated, at Time 0, with 50 nM 17β-estradiol (E2) for 24 hours. **B)** Quantification of Western blot signal intensity in panel B. **C)** T47D and MCF7 cells were treated with 100 nM ICI 182,780 (ICI), an estrogen receptor antagonist, for 24 hours.

GATA3 is tied to ERα through a positive cross-regulatory loop [14] and ERα turnover by the proteosome is intimately connected to ligand binding [28]. As GATA3 protein stability was regulated in a manner similar to ERα, we hypothesized that its stability might also be influenced by estradiol and that the frameshift mutation might impact protein-level regulation of GATA3 by hormone. Addition of estradiol to hormone starved cells results in cyclic variation of ERα levels, we chose a time point at which this cycling has stabilized (24 hours). Treating hormone-starved T47D or MCF7 cells with estradiol led to down-regulation of ERα, as expected (Figure 4A, B). GATA3 abundance in T47D cells was dramatically reduced by estradiol, mirroring ERα. In contrast, both wild type and truncated GATA3 in MCF7 were only moderately affected by hormone. FOXA1, an essential determinant of ERα expression [30] and a frequent binding partner of ERα and GATA3 [17,31], decreased in abundance following estradiol treatment in T47D, although not to the same extent as GATA3 and ERα (Figure 4A, B). In MCF7, hormone had little to no impact on FOXA1 levels. These results suggest that the stability of three DNA binding transcription factors integral to the transcriptional response to estrogen in luminal breast cancer cells, exhibits altered turnover downstream of estrogen upon mutation of one allele of *GATA3*.

Because the truncating mutation alters GATA3 protein level following hormone treatment, we asked whether the action of estrogen antagonists was likewise affected by this mutation. We treated cells grown in normal conditions (media plus FBS) with the ERα antagonist, ICI 182,780 (ICI). As expected, ERα expression was reduced in both cell lines (Figure 4C). While wild-type GATA3 protein levels were reduced following antagonist treatment in both T47D and MCF cells, the level of mutated GATA3 in MCF7 cells did not change. FOXA1 expression was not affected by ICI (Figure 4C). The GATA3 mRNA level remained mostly unaffected in cells treated with estradiol or ICI (Additional file 1: Figure S2). These experiments demonstrate that the truncation mutation in GATA3 stabilizes the protein in the face of agonist or antagonist binding by ERα, thus uncoupling physiologic, protein-level regulation from estradiol action.

### DNA binding ability of mutated GATA3 is impaired

**Figure 5:**
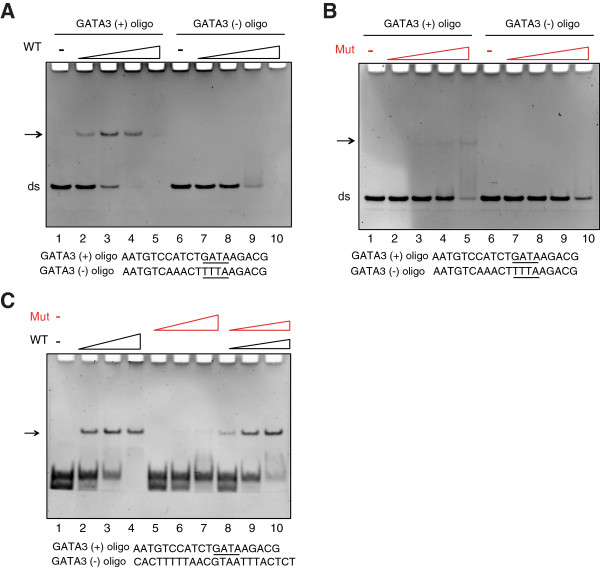
**DNA binding ability of wild-type and mutated GATA3.** Electrophoretic Mobility Shift Assay (EMSA) was performed using recombinant **A)** wild-type GATA3 DNA binding domain (DBD) or **B)** mutated GATA3-DBD. Equivalent molar amounts of wild-type and mutant protein, assessed by Coomassie blue stained SDS-PAGE, were used. **C)** In a competitive assay, an equimolar mix of wild-type and mutated DBD proteins was used. To test protein ability to bind DNA, GATA3 motif-containing oligonucleotides (GATA +) and GATA3 motif-lacking oligonucleotides (GATA-) were used. WT – wild-type, Mut – mutated GATA3.

The frameshift mutation present in GATA3 in MCF7 affects the second zinc finger, which is responsible for DNA binding [32]. We employed electrophoretic mobility shift assays (EMSA) to interrogate the DNA binding capacity of mutated GATA3 protein. By titrating the recombinant DNA binding domain (DBD) of wild-type GATA3 [32], we demonstrated a shift from the free GATA-motif-containing oligonucleotide to a specific, protein bound complex (Figure 5A, lanes 1–5). In contrast, the mutant GATA3 DBD was able to bind DNA to a lesser degree and only at high concentrations of recombinant protein (Figure 5B, lanes 1–5). To assess whether the wild-type and mutant DBD protein fragments could heterodimerize on DNA, we mixed recombinant wild-type and mutated DBDs in a competitive assay with GATA motif-containing and GATA motif-lacking oligonucleotide. We did not observe any evidence of a complex with altered mobility following addition of mutant GATA3 DBD (Figure 5C, lanes 1–4 and 8–10). A similar experiment without competitor DNA had identical results (data not shown).

**Figure 6:**
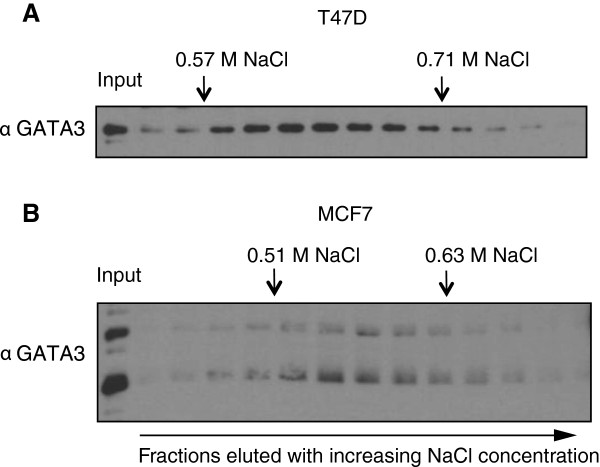
**Biochemical characterization of wild-type and mutant GATA3 using a nucleic acid analog.** Nuclear extracts were prepared from **A)** T47D and **B)** MCF7 cells and applied to heparin sepharose. The column was eluted with linear gradient of NaCl concentration from 0.1 to 1 M. The separated fractions were analyzed by Western blot directed against GATA3.

To further characterize the ability of endogenous GATA3 to bind DNA, we utilized heparin, a glycosaminoglycan structurally similar to nucleic acids. Nuclear extracts obtained from T47D and MCF7 cells were partially purified through ion exchange chromatography, applied to a heparin column, and eluted with a linear gradient of NaCl. The peak of full-length GATA3 from T47D eluted between 0.57 and 0.71 M NaCl (Figure 6A). In MCF7 both full-length and truncated GATA3 were eluted in the same range of salt, 0.51-0.63 M (Figure 6B), indicating a potential for formation of GATA3 wild-type/mutant heterodimers under these conditions.

### Genomic location of GATA3 in breast cancer cells

**Figure 7:**
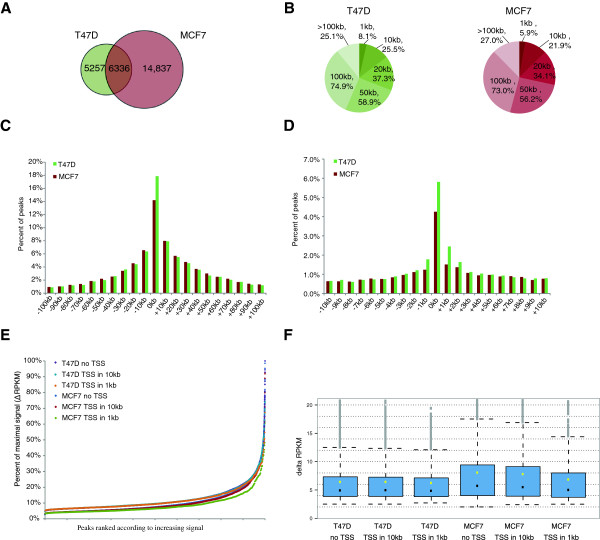
**Genomic location of wild-type and mutated GATA3 transcription factor in T47D and MCF7 cell lines. A)** Number of GATA3 ChIP-seq peaks in T47D and MCF7 cells. **B)** GATA3 peak distribution relative to distance from the closest transcription start site (TSS) in T47D and MCF7 cells. **C)** GATA3 ChIP-seq peak distribution within 10 kb from the closest TSS in T47D and MCF7 cells. **D)** GATA3 ChIP-seq peak distribution within 100 kb from the closest TSS in T47D and MCF7 cells. **E)** GATA3 occupancy enriched regions sorted by increasing ChIP-seq signal. Peaks were divided into peaks not associated with a TSS (no TSS) and peaks within 1 kb or 10 kb from the closest TSS (TSS in 1 or 10 kb). y-axis is scaled to % of maximal for given cell line RPKM (reads per kilobase per million). **F)** ChIP-seq signal range in T47D and MCF7 cells. Box represents 25% to 75% of the range and whiskers are 5% to 95%. Black dot is median and yellow dot is mean value. Legend - see **E)**.

To analyze the genomic location of GATA3 transcription factor in T47D and MCF7 cells, we performed chromatin immunoprecipitation (ChIP) on asynchronous cultures. We first established a robust ChIP assay using polyclonal sera raised against full length recombinant GATA3 (which recognizes both full-length and mutant proteins). We assessed GATA3 enrichment at published positive control loci [14] using PCR for detection (Additional file 1: Figure S3). ChIP DNA was used to prepare standard libraries for massively parallel sequencing under conditions that preserve enrichment for the positive control regions (Additional file 1: Figure S4). Sequencing was performed on two biological replicates from T47D and MCF7 cells, resulting in 33-45×10^6^ reads per library. Following quality control, mapping to unique positions in the human genome, and deduplication, 37-59% reads were retained (Additional file 2: Table S1). Sequencing reads from the two libraries from each cell line were merged (Additional file 2: Table S2). The HOMER algorithm identified 11,593 enriched regions (peaks) in T47D cells and 21,173 in MCF7 cells. Total 6,336 of these peaks overlapped by at least 1 bp (Figure 7A). Selected ChIP-seq peaks were validated using Real-Time PCR (Additional file 1: Figure S5).

We explored the similarities in ChIP-seq between the two cell lines in terms of location relative to genomic features and intensity. In spite of the difference in number of GATA3 enriched regions, peak distribution relative to the closest transcription start site (TSS) was similar in T47D and MCF7 cells (Figure 7B). T47D cells had modestly higher frequency of enrichment in the range from -1 kb to +2 kb from TSS (Figure 7C, D). When the peaks were sorted by ChIP-seq signal, distribution in both cell lines was almost indistinguishable for peaks within 10 kb from the closest TSS as well as for those not associated with TSS – meaning that high-intensity peaks were distributed in a similar manner (Figure 7E). While these general indicators of pattern of enrichment appeared highly similar across the two cell lines, we observed a difference in overall signal intensity at peaks. Regardless of their localization relative to TSSs, bins of peaks in MCF7 exhibited a broader range of signal intensity than comparable bins in T47D (Figure 7F). This relationship was further corroborated by scrutiny of mean and median values for peaks grouped according to distance from TSS, where MCF7 invariably had higher mean and median values. At a gross level, the overall pattern of association of GATA3 with the genome appeared highly similar between the two cell lines. However, peak signal intensity after normalization for read depth was higher in MCF7 than in T47D.

Assigning GATA3 peaks to the closest transcription start site (TSS) resulted in 4524 genes within 50 kb in T47D and 6934 in MCF7, including 3011 genes overlapping between both cell lines (Table 1, Additional file 3: Tables S3 and S4). Ingenuity Pathway Analysis of genes associated with genomic regions enriched for GATA3 binding indicated that cell cycle, death and survival, growth and proliferation, movement, and development were the top functional categories related to GATA3 presence in T47D cells (Additional file 1: Figure S6). The top molecular and cellular functions in MCF7 were similar and included cellular growth and proliferation, development, movement, death and survival, and cell to cell signaling and interaction (Additional file 1: Figure S7). A more detailed analysis focusing on genes involved in mammary tissue differentiation, breast cancer subtype determination, or estrogen response [31,33-35], showed that although GATA3 was present near 20-60% of TSSs, there was no enrichment detected when compared to the expected occupancy in a given functional group. Also, there were no major differences in the tested datasets between GATA3 transcription factor binding in T47D and MCF7 cells (Additional file 1: Figures S8-S10).

**Table 1:** Number of genes associated with GATA3 transcription factor binding in T47D and MC7 breast cancer cell lines within 10 and 50 kb from the closest transcription start site (TSS)

**Cell line**	Number of genes^*^	
	**+/−10 kb from TSS**	**+/−50 kb from TSS**
T47D	2433	4524
MCF7	3592	6934
Common	1649	3011

^*^Genes may contain more than one GATA3 peak within specified distance from TSS.

**Figure 8:**
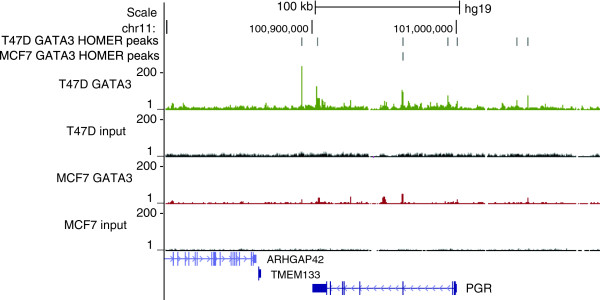
Transcription factor GATA3 binding at progesterone receptor (PGR) locus in T47D and MC7 cells.

There were, however, individual genes that differed substantially in GATA3 enrichment between cell lines (Additional file 4: Table S5). The progesterone receptor (PGR) is an example of an individual locus displaying a difference in GATA3 occupancy between cell lines, with T47D featuring seven peaks spanning over 200 kb and MCF7 cells containing only one peak in the same region (Figure 8). The biologic significance of this finding remains unclear, although the reduced expression of PGR in MCF7, as compared to T47D, may be reflective of alterations in GATA3 binding (Additional file 1: Figure S11).

Using a mobility shift assay, we established that the frameshift mutation present in *GATA3* in MCF7 impairs the protein’s ability to bind DNA. To test whether the mutation affects the capacity to specifically recognize DNA sequence in the genome, we evaluated the frequency of occurrence of the GATA3 canonic recognition motif, WGATAR within the ChIP-seq peaks. The proportion of GATA3 peaks containing the recognition motif in T47D and MCF7 cells was essentially identical - 71.4% and 72.7%, respectively (Table 2). We explored whether binning peaks in several different ways impacted this similarity and found that no matter how we grouped the peaks, the frequency of peaks containing the consensus GATA3 element was consistent between the two cell lines (Additional file 1: Figure S12). These data suggest that the mutation in MCF7 does not significantly change the binding site preference of GATA3 in the context of chromatin.

**Table 2:** Frequency of GATA3 recognition motif WGATAR in GATA3-peaks identified by ChIP-seq in T47D and MCF7 cells

**GATA3 peaks**	**All**	**Peaks containing WGATAR motif**	
		**Number**	**Percent [%]**
T47D	11593	8281	71.4
T47D peaks overlapping with MCF7	6336	4655	73.5
T47D-specific	5257	3626	69.0
MCF7	21173	15392	72.7
MCF7 peaks overlapping with T47D	6336	4590	72.4
MCF7-specific	14837	10802	72.8

## Discussion

Large-scale genome sequencing projects have provided, and continue to provide, volumes of information on the mutational landscape of cancers. A current challenge for cancer biologists is to investigate the emerging genomic data in a mechanistic context, establishing the relationship of specific mutations to tumor biology and informing on clinical parameters including aggressiveness, response to therapy, and potential for metastasis. Here, we have initiated an attempt to address the mechanistic basis by which mutations in the transcription factor *GATA3* may provide a growth advantage to breast cancer cells. The Cancer Genome Atlas Network (TCGA) recently reported a comprehensive study of human breast cancer: tumors from 507 patients were analyzed on multiple high information content platforms: whole exome sequencing, DNA copy number arrays, DNA methylation, mRNA array and sequencing, microRNA sequencing and reverse-phase protein arrays [1]. Somatic mutations in *GATA3* occurred in 58 cases (10.7%), predominantly in luminal A and B cancer subtypes, an additional 12 samples displayed copy number alterations (http://www.cbioportal.org). Strikingly, while mutations of *GATA3* in the congenital disorder HDR syndrome are found throughout the protein [22], breast cancer specific mutations occur almost exclusively in exons 5 and 6 (TCGA). This clustering suggests regulatory roles for the carboxyl terminus of GATA3 and that impairment of these functions can provide a growth advantage to cancer cells.

Careful scrutiny of the TCGA mutation data revealed that six mutations were localized in the second zinc finger and five of them were frameshifts, similar to the mutation in MCF7 [23], making MCF7 a useful model to study a clinically relevant phenomenon. We confirmed the presence of a heterozygous guanine insertion in the fifth exon of *GATA3* in the MCF7 genome and showed that although both full-length and truncated proteins were expressed, the mutated protein was present in the cells at a higher level. The D336 frameshift does not affect the N-terminal and C-terminal sequences flanking ZnF1 that are required for nuclear localization [22] and GATA3 proteins localized to the nucleus of MCF7 cells. Mutations in GATA3 ZnF2 impair DNA binding [20-22] suggesting that the same effect could be expected for MCF7-specific mutation. The biochemical fractionation assay identified a pool of truncated protein very loosely associated with chromatin (Figure 2B). However, the gel shift assay demonstrated that truncated GATA3 could bind DNA selectively, albeit with decreased affinity compared to wild-type (Figure 5). Consistent with the documented capacity of GATA3 to self-associate and to dimerize on DNA [32], we observed a pool of mutant protein that exhibited similar chromatin binding properties to wild-type GATA3. The data are consistent with formation of heterodimers between mutant and wild-type GATA3, potentially altering the association of the protein with its recognition elements in the genome.

ChIP-seq was utilized to assess the degree of overlap of GATA3 across the two cell lines used in our study. Surprisingly, the number of binding sites detected in MCF7 was substantially higher than in T47D cells. In spite of the large difference in genomic occupancy, detailed analysis of genes associated with GATA3 binding failed to identify any major functional differences between binding profiles in T47D and MCF7 cell lines (Additional file 1: Figure S6-S10). We speculated that the increased number of GATA3-enriched regions in MCF7 genome could have been due to compromised ability of the truncated protein to recognize the specific GATA binding motif, WGATAR. However, the proportion of GATA3 peaks containing the WGATAR motif was nearly identical in binding regions identified in T47D and MCF7 cells, as well as in cell-line specific regions (Table 2). This finding suggested that the heterozygous mutation did not affect binding specificity in MCF7 cells.

Although the number of GATA3 peaks was considerably lower in T47D than in MCF7 cells, progesterone receptor gene was an example of a locus featuring a greater number of bound regions in T47D than in MCF7. Remarkably, lack of PGR expression, as determined by immunohistochemical staining, was a common denominator for all five patients in the TCGA database carrying a frameshift mutation in ZnF2 of GATA3 (http://www.cbioportal.org). Even though both T47D and MCF7 cell lines are classified as PGR and ERα positive, and belong to luminal A breast cancer subtype [36], MCF7 has been also used as a model for luminal B subtype [37]. The luminal B subtype is the more aggressive form of ERα-positive breast cancer that is less responsive to endocrine therapy [38]. It is characterized by increased expression of proliferation-related genes and lower expression of ER-dependent genes, including PGR [38,39]. In our model system, PGR mRNA level was approximately 20-fold lower in MCF7 than in T47D cells (Additional file 1: Figure S11). Loss of PGR expression is often considered as a marker for the gain of hormone-independent growth properties by ERα-positive breast cancers, through increased cross-talk between ERα and growth factor signaling pathways [38,40]. In addition, the normal balance of the two known PGR isoforms, A or B, impacts biological properties of tumors [41].

Comparison of the biochemical properties of mutated GATA3 with wild type protein present in the T47D cell line demonstrated an increased half-life of truncated GATA3 in normal growth conditions and in response to ERα agonist and antagonist (Figures 3 and 4). GATA3 levels were proteasome-dependent (Figure 3B), similar to ERα, where rapid turnover of the receptor upon ligand binding is based on the ubiquitin-proteasome pathway [28]. GATA3 is required for estrogen stimulation of cell cycle progression in breast cancer cells [14] and we showed that this truncating mutation present in MCF7 genome uncouples protein level regulation from hormonal signaling.

## Conclusions

These findings strongly suggest that the carboxyl terminus of GATA3, a mutational hotspot in breast cancer, confers regulation on protein levels through as yet undefined mechanisms, resulting in increased stability of transcription factors resident on critical response elements in the breast cancer genome. We predict that mutations in GATA3 with similar characteristics to the mutation in MCF7 likely confer a growth advantage, particularly in pre-menopausal women, and are likely to occur early in tumor evolution.

## Competing interest

The authors declare no competing interest.

## Authors’ contribution

Experimental design – ABA, MT, JKS, PAW. Performed experiments – ABA, CM, MT, JKS. Data analysis – SAG, ABA, PAW. Manuscript preparation – all authors. All authors read and approved the final manuscript.

## Pre-publication history

The pre-publication history for this paper can be accessed here:

http://www.biomedcentral.com/1471-2407/14/278/prepub

## Supplementary Material

Additional file 1: Figure S1 Quantification of Western blot signal intensity for full-length and truncated GATA3 protein in T47D and MCF7 cells treated with DMSO (Vehicle) or cycloheximide (CHX) over the course of eight hours. **Figure S2.** GATA3 mRNA level in T47D and MCF7 cells treated with estradiol (E2) or ICI 182,780 (ICI). **Figure S3.** GATA3 enrichment determined by Real-Time PCR in ChIP reactions pooled for ChIP-seq library preparation. **Figure S4.** GATA3 enrichment in PCR-amplified ChIP-seq library in positive (ESR1 Enh1) and negative (ESR1 -6.9 kb) control regions located upstream from estrogen receptor transcription start. **Figure S5.** Real-Time PCR validation of GATA3 binding in T47D and MCF7 genome determined by ChIP-seq. **Figure S6.** Functional classification of genes located within 50 kb from a GATA3 ChIP-seq peak in T47D cells. **Figure S7.** Functional classification of genes located within 50 kb from a GATA3 ChIP-seq peak in MCF7 cells. **Figure S8.** GATA3 ChIP-seq peak presence in T47D and MCF7 cells within 10 kb from TSS of genes involved in normal mammary cell commitment and differentiation. **Figure S9.** GATA3 ChIP-seq peak presence in T47D and MCF7 cells within 50 kb from TSS of estrogen-responsive genes. **Figure S10.** GATA3 ChIP-seq peak presence in T47D and MCF7 cells within 50 kb from TSS of genes differentially expressed in different molecular subtypes of breast cancer. **Figure S11.** Progesterone receptor (PGR) expression in T47D and MCF7 cells treated with GATA3 siRNA (Error bars represent standard deviation; n=2) or infected with GATA3 adenovirus (Adv) (n=1). **Figure S12.** Frequency of GATA3 recognition motif, WGATAR, in GATA3 ChIP-seq peaks located within 10 kb. Click here for file

Additional file 2: Table S1 Number of reads in each sequencing run for GATA3 ChIP-seq in T47D and MCF7 cell lines. **Table S2.** Number of reads in merged samples for two replicates of GATA3 ChIP-seq in T47D and MCF7 cell lines. Click here for file

Additional file 3: Table S3 GATA3 ChIP-seq peaks identified in T47D cell line. Peaks overlapping with MCF7 by at least 1 bp are indicated, as well as the nearest gene to the peak, within 10 kb or 50 kb from peak border. **Table S4.** GATA3 ChIP-seq peaks identified in MCF7 cell line. Peaks overlapping with T47D by at least 1 bp are indicated, as well as the nearest gene to the peak, within 10 kb or 50 kb from peak border. Click here for file

Additional file 4: Table S5 Genes with GATA3 ChIP-seq peak within 10 kb from transcription start site (TSS). Click here for file
