# Supplementary material for: Breast tumor specific mutation in GATA3 affects physiological mechanisms regulating transcription factor turnover
Source: BMC Cancer. 2014 Apr 22;14:278. doi: 10.1186/1471-2407-14-278 (PMC4021073; doi:10.1186/1471-2407-14-278)
Supplement: Additional file 1: Figure S1 — Quantification of Western blot signal intensity for full-length and truncated GATA3 protein in T47D and MCF7 cells treated with DMSO (Vehicle) or cycloheximide (CHX) over the course of eight hours. Figure S2. GATA3 mRNA level in T47D and MCF7 cells treated with estradiol (E2) or ICI 182,780 (ICI). Figure S3. GATA3 enrichment determined by Real-Time PCR in ChIP reactions pooled for ChIP-seq library preparation. Figure S4. GATA3 enrichment in PCR-amplified ChIP-seq library in positive (ESR1 Enh1) and negative (ESR1 -6.9 kb) control regions located upstream from estrogen receptor transcription start. Figure S5. Real-Time PCR validation of GATA3 binding in T47D and MCF7 genome determined by ChIP-seq. Figure S6. Functional classification of genes located within 50 kb from a GATA3 ChIP-seq peak in T47D cells. Figure S7. Functional classification of genes located within 50 kb from a GATA3 ChIP-seq peak in MCF7 cells. Figure S8. GATA3 ChIP-seq peak presence in T47D and MCF7 cells within 10 kb from TSS of genes involved in normal mammary cell commitment and differentiation. Figure S9. GATA3 ChIP-seq peak presence in T47D and MCF7 cells within 50 kb from TSS of estrogen-responsive genes. Figure S10. GATA3 ChIP-seq peak presence in T47D and MCF7 cells within 50 kb from TSS of genes differentially expressed in different molecular subtypes of breast cancer. Figure S11. Progesterone receptor (PGR) expression in T47D and MCF7 cells treated with GATA3 siRNA (Error bars represent standard deviation; n=2) or infected with GATA3 adenovirus (Adv) (n=1). Figure S12. Frequency of GATA3 recognition motif, WGATAR, in GATA3 ChIP-seq peaks located within 10 kb. [file 1471-2407-14-278-S1.pdf]

Fig S1

A

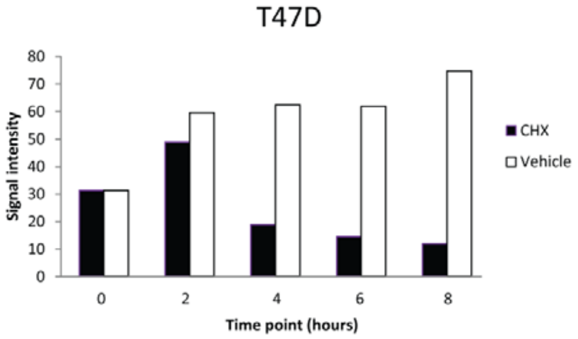

B

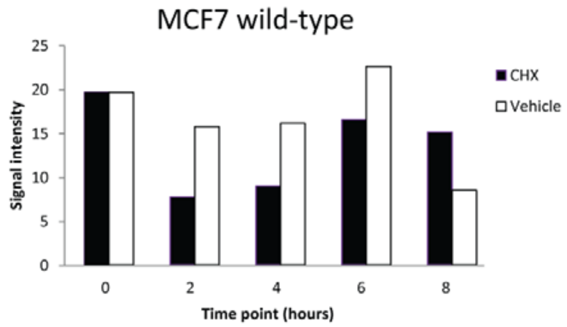

C

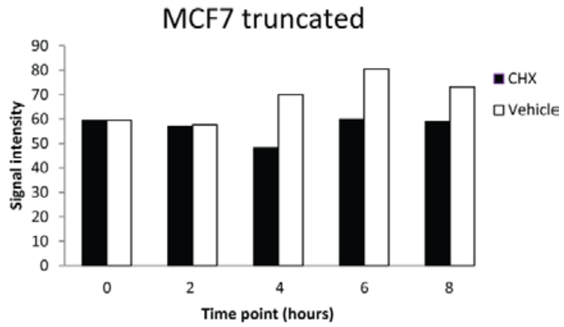

Figure S1. Quantification of Western blot signal intensity for full-length and truncated GATA3 protein in A) T47D and B, C) MCF7 cells treated with DMSO (Vehicle) or cycloheximide (CHX) over the course of eight hours (see Fig. 3). Quantification was performed with Quantity One software (Bio-Rad), using volume tool.

Fig S2

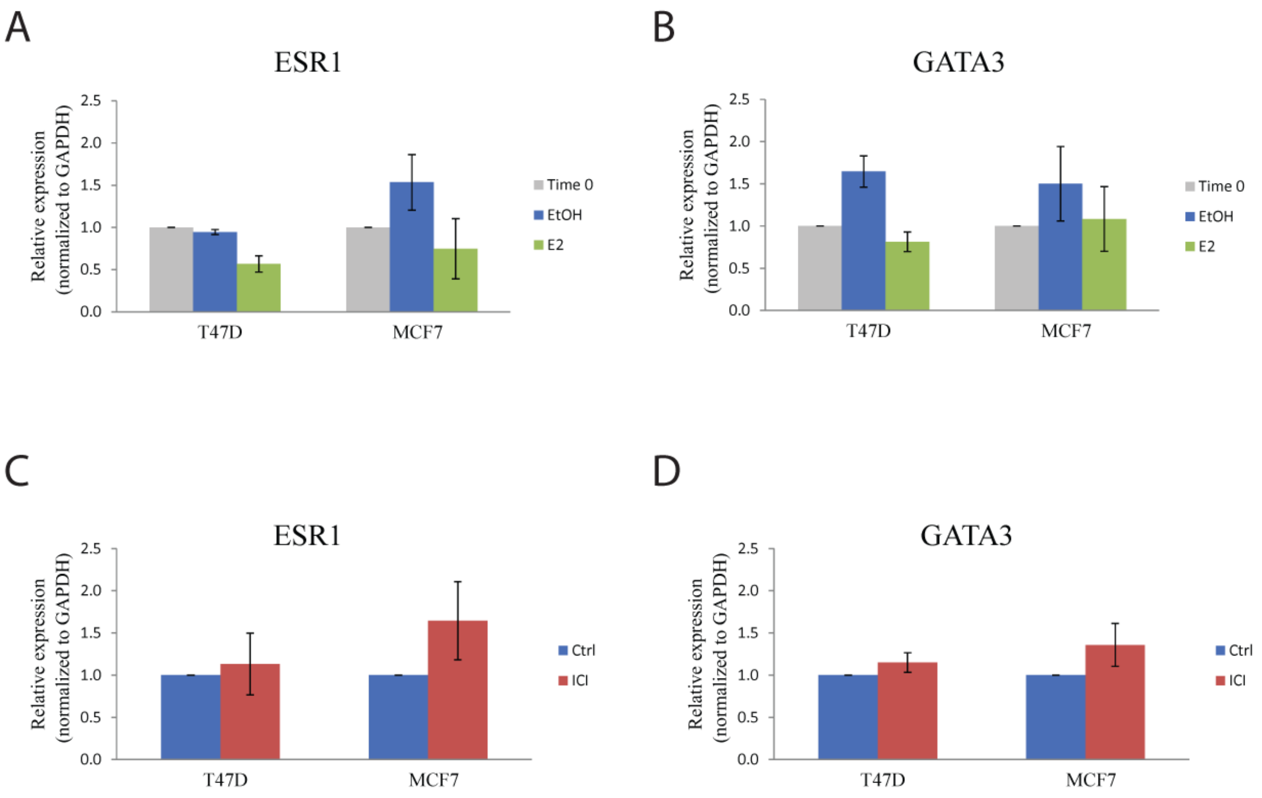

Figure S2. GATA3 mRNA level in T47D and MCF7 cells treated with A, B) estradiol (E2) or C, D) ICI 182,780 (ICI) (see Fig. 4). Error bars represent standard deviation, n=2.

Fig S3

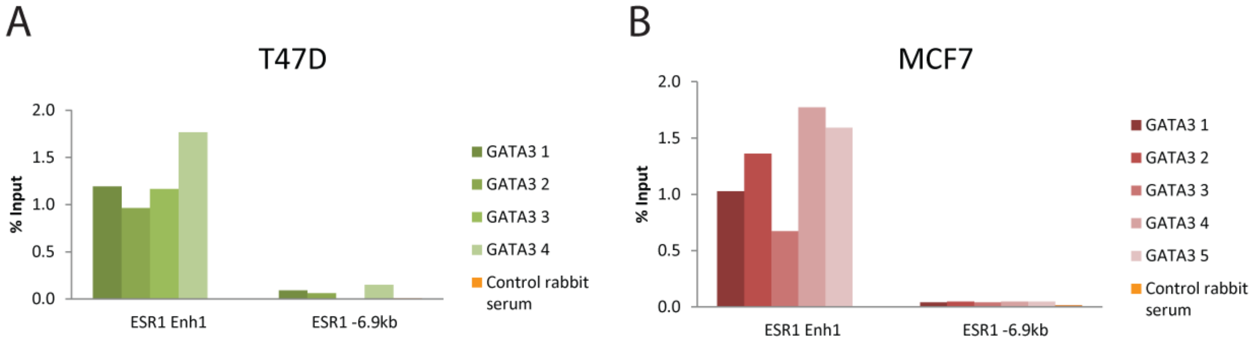

C

ESR1 Enh1_F	TGTAGGCTAGTTTTGTTTAACGATTTTT
ESR1 Enh1_R	GGTGATGGGAGAATTGCTTAGAA
ESR1 -6.9kb_F	TCTGTCCTCCTCGTTCTACCTCC
ESR1 -6.9kb_R	AACAACAGCGATTTATTTTCTCAGTG

Figure S3. GATA3 enrichment determined by Real-Time PCR in ChIP reactions pooled for ChIP-seq library preparation. A) Four reactions were pooled for T47D, and B) five for MCF7 ChIP-seq. C) Positive (ESR1 Enh1) and negative (ESR1 -6.9kb) control regions were located upstream from estrogen receptor transcription start site [14]. Shawn samples were used for the first ChIP-seq replicate.

Fig S4

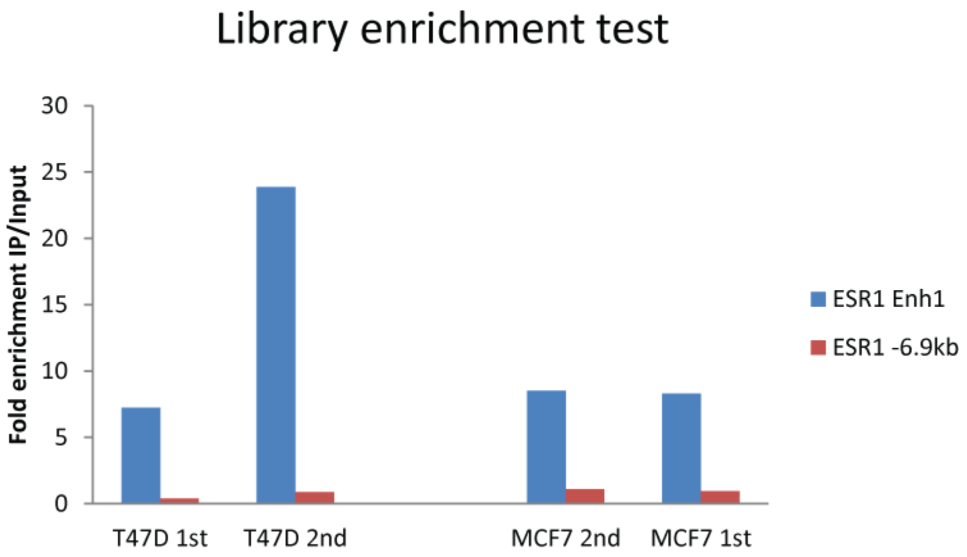

Figure S4. GATA3 enrichment in PCR-amplified ChIP-seq library in positive (ESR1 Enh1) and negative (ESR1 -6.9kb) control regions located upstream from estrogen receptor transcription start [14]. 1st – first ChIP-seq replicate, 2nd – second ChIP-seq replicate.

Fig S5

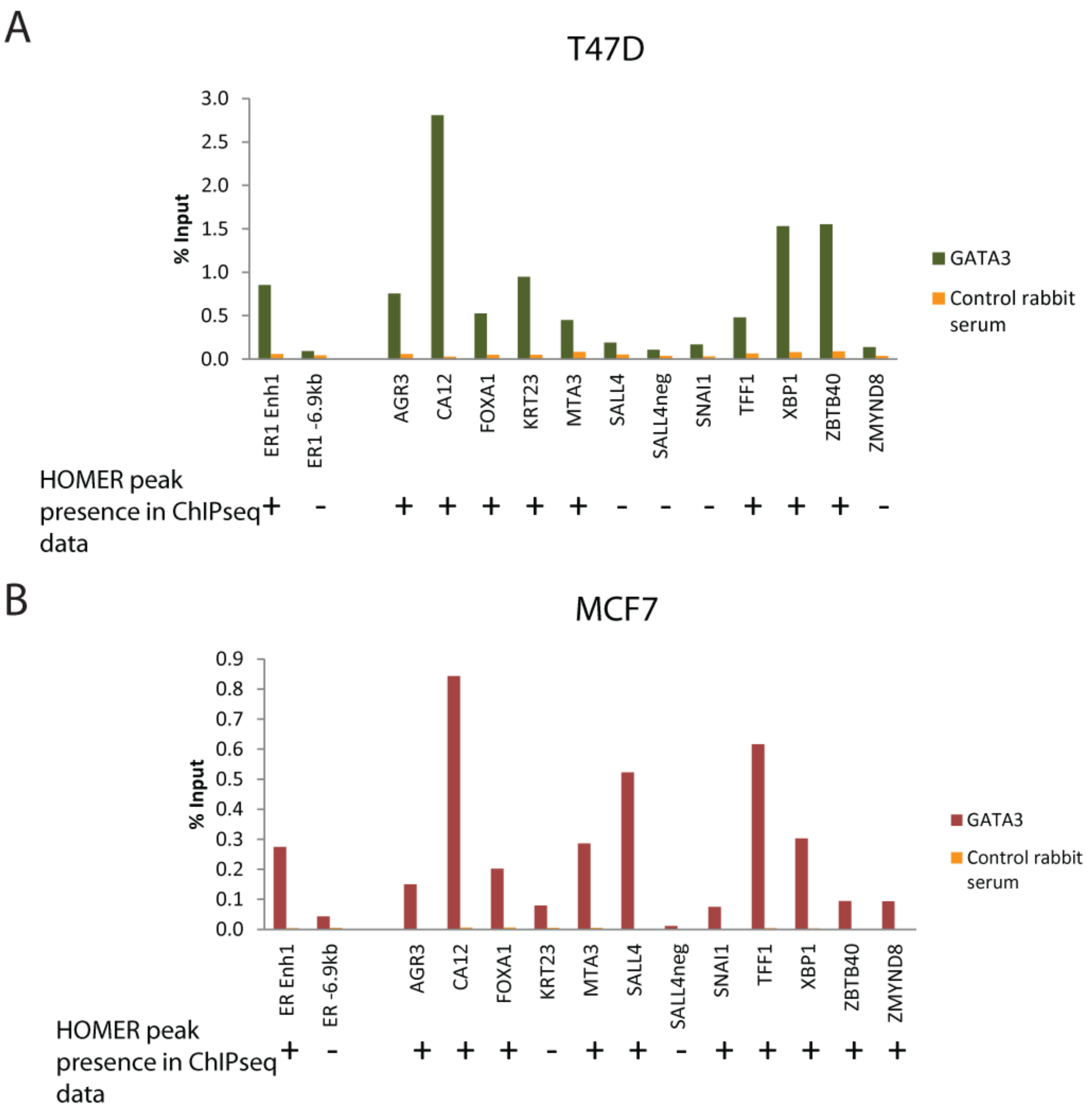

Figure S5. Real-Time PCR validation of GATA3 binding in A) T47D and B) MCF7 genome determined by ChIP-seq.

Fig S6

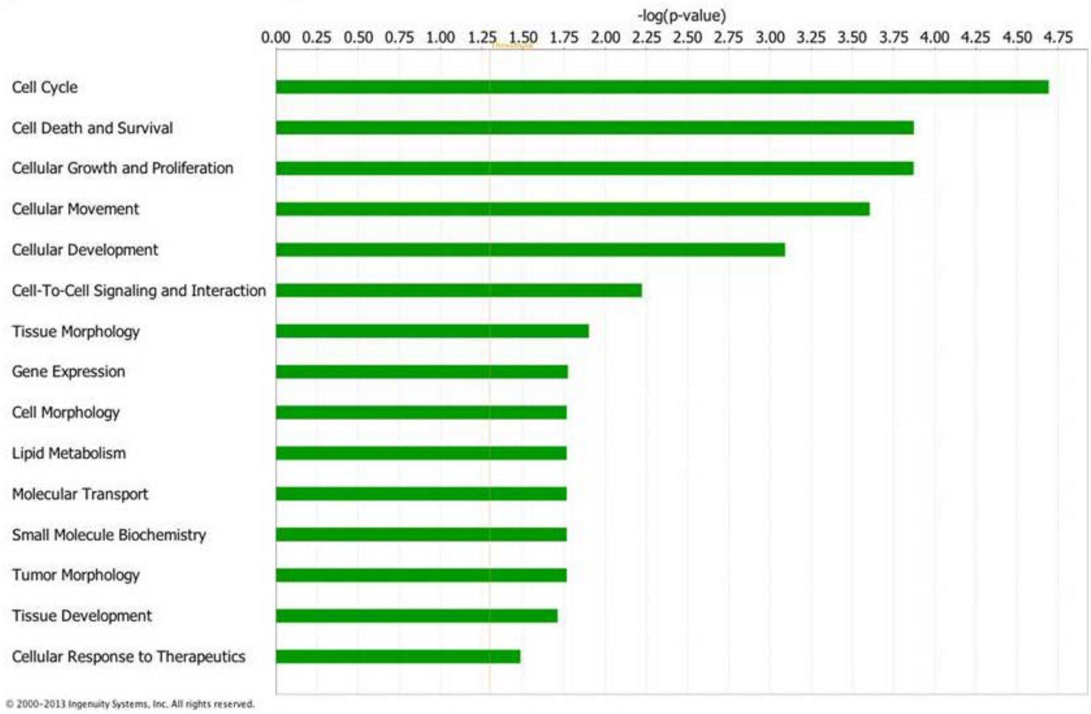

Figure S6. Functional classification of genes located within 50kb from a GATA3 ChIP-seq peak in T47D cells. The bar chart was generated using IPA application (Ingenuity Systems, Redwood City, CA, USA). The analysis was restricted to relationships relevant in human mammary gland and breast cancer cell lines. Genes associated with GATA3 peaks in T47D that the analysis was based on are provided in Supplementary Table S3.

Fig S7

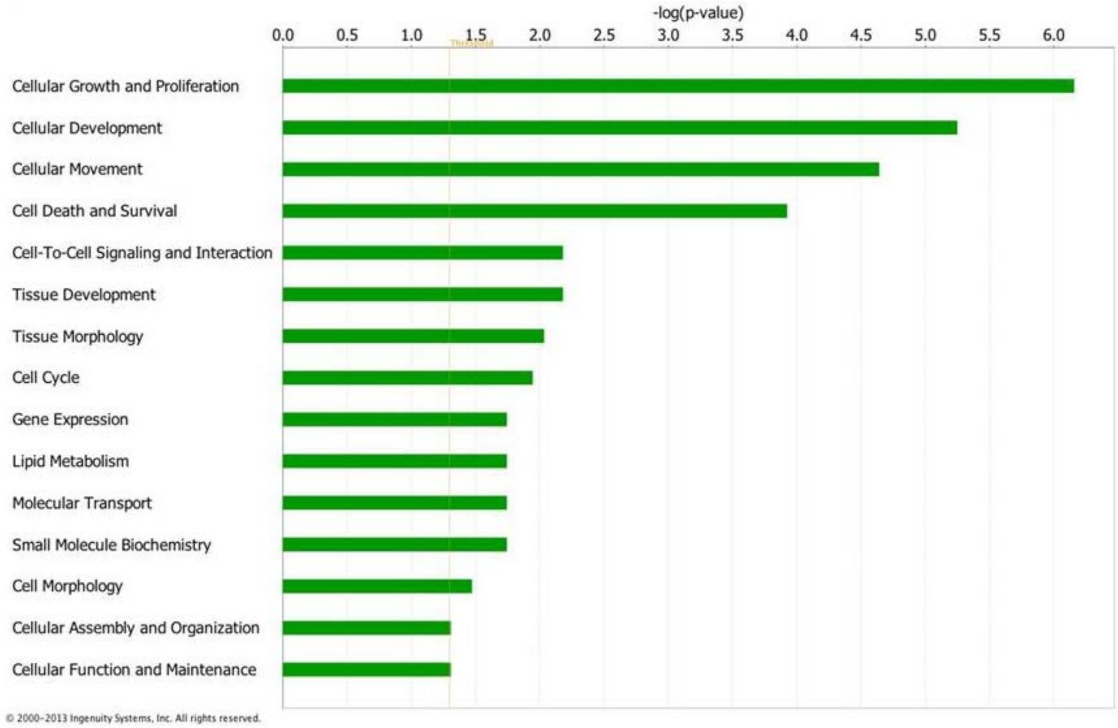

Figure S7. Functional classification of genes located within 50kb from a GATA3 ChIP-seq peak in MCF7 cells. The bar chart was generated using IPA application (Ingenuity Systems, Redwood City, CA, USA). The analysis was restricted to relationships relevant in human mammary gland and breast cancer cell lines. Genes associated with GATA3 peaks in MCF7 that the analysis was based on are provided in Supplementary Table S4.

Fig S8

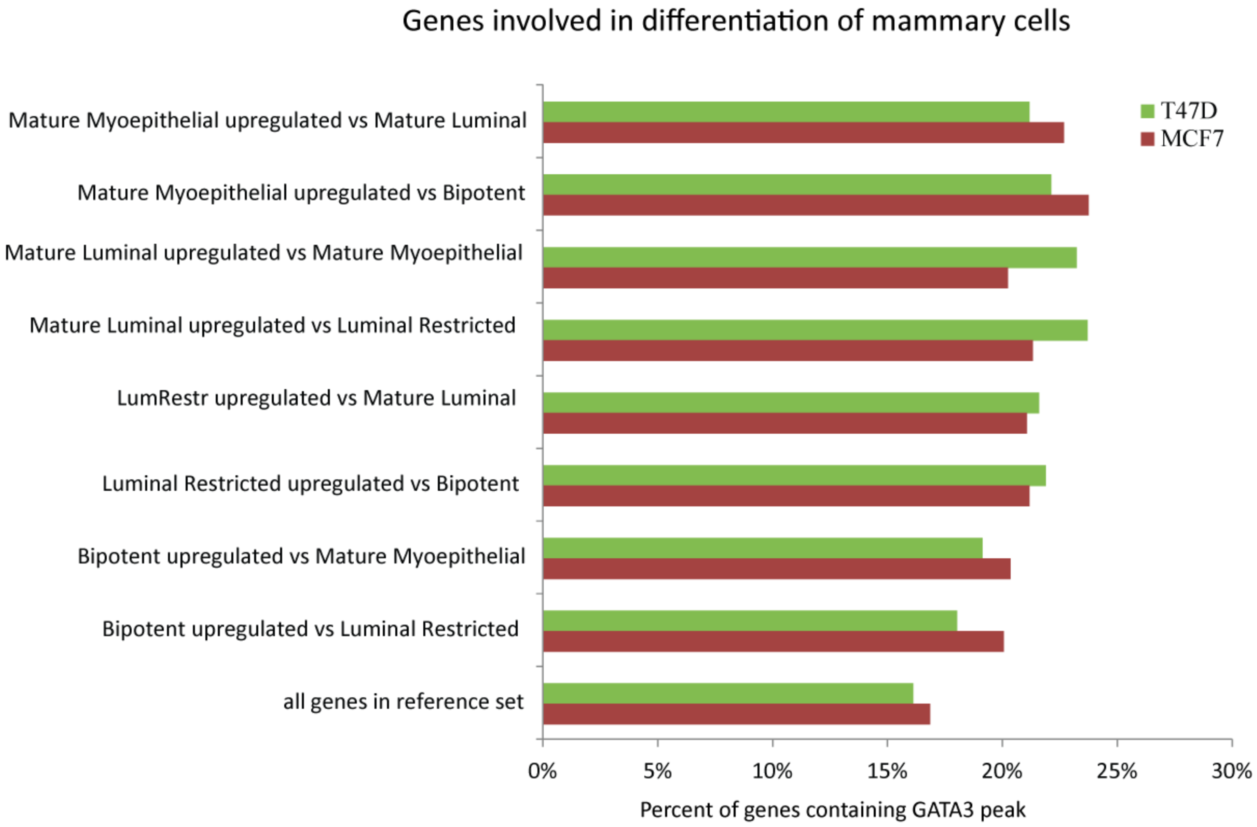

Figure S8. GATA3 ChIP-seq peak presence in T47D and MCF7 cells within 10kb from TSS of genes involved in normal mammary cell commitment and differentiation. Genes differentially expressed in bipotent, luminal restricted, mature myoepithelial or mature luminal cells were obtained from Raouf et al. [33]. There were between 250 and 520 genes in each analyzed category.

Fig S9

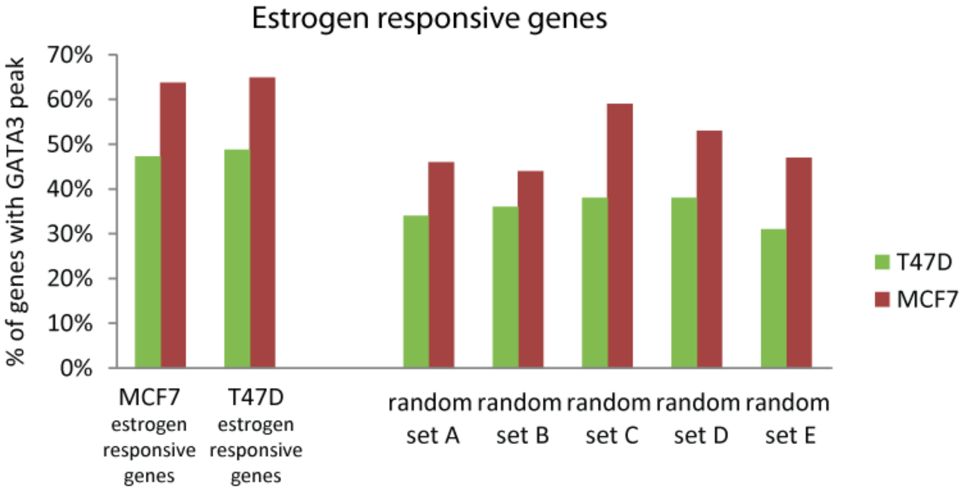

Figure S9. GATA3 ChIP-seq peak presence in T47D and MCF7 cells within 50kb from TSS of estrogen-responsive genes. Genes differentially expressed in response to estradiol in T47D (322 genes) were obtained from Lin et al. [34] and in MCF7 (1458 genes) from Carroll et al. [31]. Each random gene set contains 100 randomly selected genes.

Fig S10

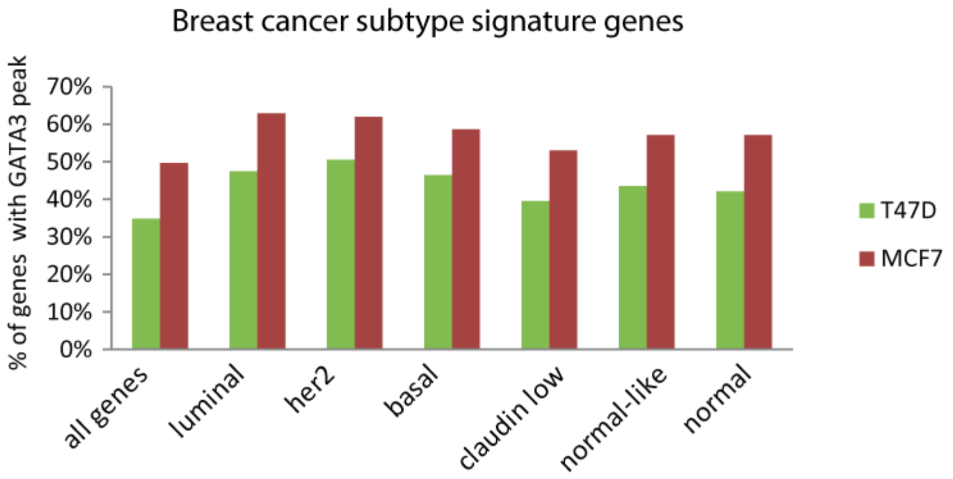

Figure S10. GATA3 ChIP-seq peak presence in T47D and MCF7 cells within 50kb from TSS of genes differentially expressed in different molecular subtypes of breast cancer. Breast cancer signature genes were obtained from Prat et al. [35]. There were between 509 and 1447 genes in the analyzed signature groups.

Fig S11

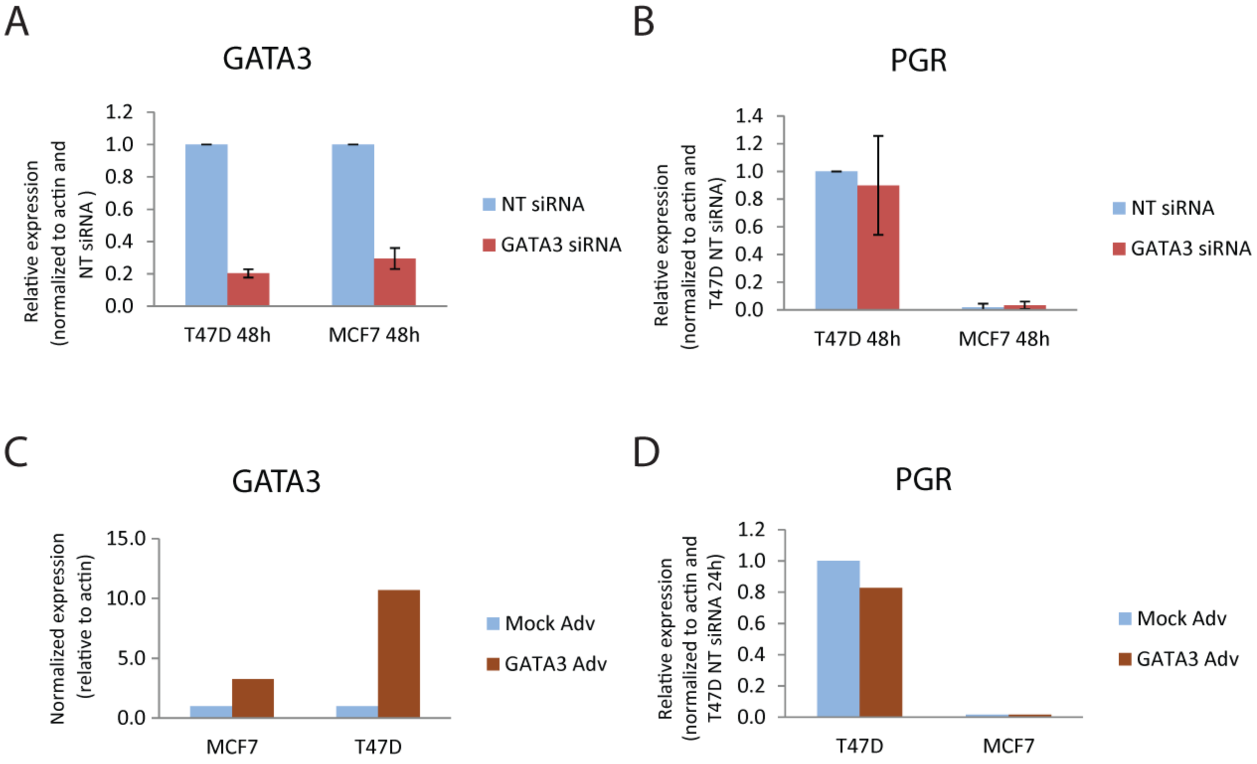

Figure S11. Progesterone receptor (PGR) expression in T47D and MCF7 cells treated with A, B) GATA3 siRNA (Error bars represent standard deviation; n=2) or C, D) infected with GATA3 adenovirus (Adv) (n=1).

Fig S12

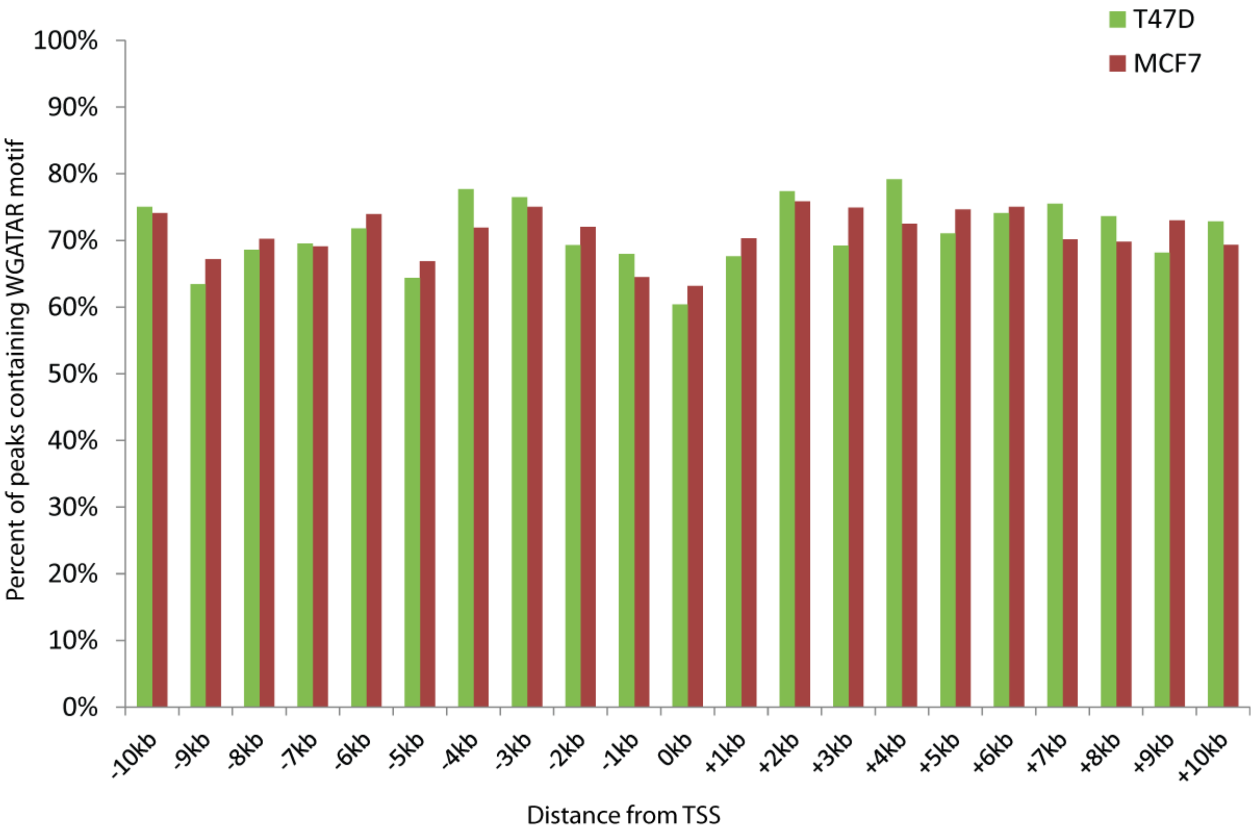

Figure S12. Frequency of GATA3 recognition motif, WGATAR, in GATA3 ChIP-seq peaks located within 10kb from the closest transcription start site (TSS).
